# Ophthalmic artery Doppler: reference values in low-risk pregnant women

**DOI:** 10.1007/s00404-026-08489-x

**Published:** 2026-07-01

**Authors:** Ladina Campana-Rüegg, Martina Hofmann, Julia Wawrla-Zepf, Ruth von Mering, Markus Gonser, Nicole Ochsenbein-Kölble, Ladina Vonzun

**Affiliations:** 1 Department of Obstetrics, University Hospital Zurich, Frauenklinikstrasse 10, 8091 Zurich, Switzerland; 2 University of Zurich, Rämistrasse 71, 8091 Zurich, Switzerland; 3 The Zurich Center for Fetal Diagnosis and Therapy, University of Zurich, Zurich, Switzerland; 4 Obstetric Research Unit, University of Zurich, Schmelzbergstrasse 12, 8091 Zurich, Switzerland; 5 Prenatal Medicine and Sonography, 72072 Tübingen, Germany

**Keywords:** Ophthalmic artery Doppler, Reference values, Preeclampsia, Hemodynamics, Vasoconstriction, Pulse wave

## Abstract

**Introduction:**

Ophthalmic artery (OA) Doppler has been described as an independent parameter in predicting preeclampsia (PE). Considering that vasoconstriction occurs in the pathogenesis of PE and causes increased pulse wave reflection, it is not surprising that OA waveform changes appear in PE. It was found that of these waveform changes, the 2nd systolic peak (P2) and the peak ratio [PR = P2/P1 (P1 = 1st systolic peak)] showed the best performance in prediction and complementary diagnosis of PE. The aim of this study was to determine reference values for OA-Doppler indices in the 1st, 2nd, and 3rd trimesters and at term in low-risk pregnant women of a mixed European population.

**Materials/methods:**

This is a prospective single-center cohort study conducted from 2020 to 2024. Only healthy pregnant women who gave informed consent were included. Women at high risk for PE or who developed PE were excluded. OA Doppler was performed according to a strict protocol at 4 distinct gestational-week (GW) intervals: 11–14 GW, 20–24 GW, 30–34 GW, and at term. The following systolic waveform parameters were focused on: P1, P2, and PR. Reference values were generated for these waveform parameters for these study intervals in the 1st, 2nd, and 3rd trimesters and at term.

**Results:**

A total of 255 women were included in this study. Of these, 62% were Caucasian, 7% Asian, 8% Afro-Caribbean, and 33% mixed or of unknown ethnicity. In this population, the PR averaged over the study intervals was 0.56, with no significant differences between the 4 study intervals or between the right and left eyes.

**Conclusion:**

This study presents systolic OA-Doppler reference values in low-risk pregnant women of an ethnically diverse European tertiary-care obstetric population. OA-Doppler reference values are stable throughout the four selected time points in pregnancy. These findings may pave the way for further investigations on OA-Doppler values as a predictive and diagnostic tool for PE.

**Supplementary Information:**

The online version contains supplementary material available at https://doi.org/10.1007/s00404-026-08489-x.

## What does this study adds to the clinical work

**Table:** 

OA Doppler systolic waveform parameters remained stable throughout pregnancy in low-risk healthy women, showing no significant variation across the first, second, and third trimesters or at term, and no significant differences between the right and left eye. These findings establish reference values for a healthy, ethnically diverse European obstetric population	
The stable reference values established in this study provide a foundation for future research assessing the clinical utility of OA Doppler indices as markers for the prediction and complementary diagnosis of preeclampsia.	

## Introduction

Despite preeclampsia (PE) being one of the major complications in pregnancy, going along with high rates of maternal and fetal morbidity and mortality, the only causal treatment remains delivery [1]. With publication of the ASPRE Trial in 2017 [2], there is good evidence that prophylactic low-dose aspirin, started before 16 week gestation (GW), can reduce the risk of PE. Thus, early and adequate screening is an important factor in reducing the risk for PE. Screening for PE consists of a combination of maternal characteristics and risk factors, mean arterial blood pressure (MAP), uterine artery Doppler, and biochemical parameters [3–6]. The latter are not available everywhere and go along with non-negligible costs.

**Fig. 1:**
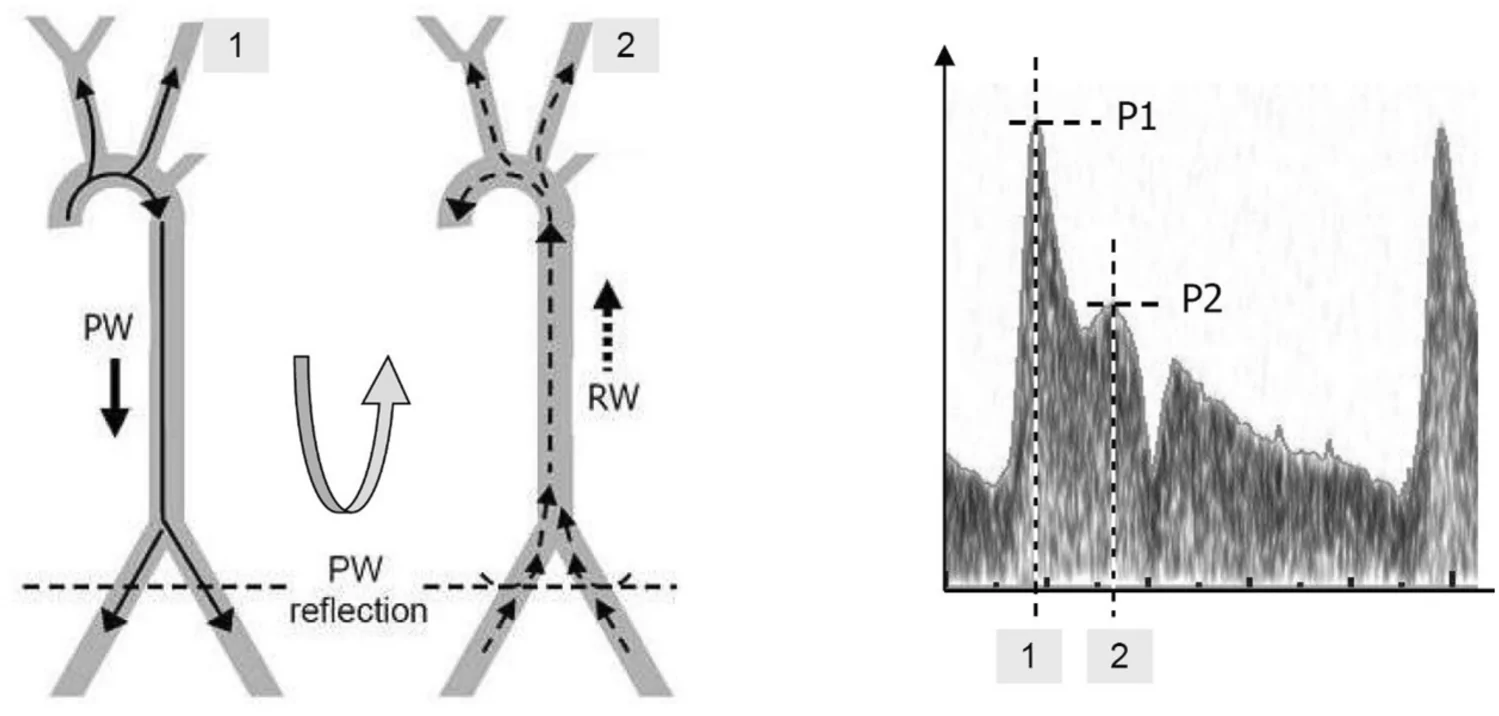
Pulse wave propagation and reflection in the arterial system and ophthalmic artery (OA) Doppler waveform. Left: schematic diagram of the arterial system showing pulse wave (PW) propagation (solid arrows), as well as reflection and return of the reflected wave (RW, dashed arrows). Thus, a PW reaches the cerebral arteries twice: by direct transmission (1) and after reflection, return, and subsequent transmission (2). Right: OA-Doppler waveform showing a biphasic systole with two peaks: P1, the main systolic velocity peak (contraction peak), and P2, the secondary velocity peak (reflection peak). Second-to-first systolic peak ratio, PR = P2/P1 ≈ 0.60

Recently, ophthalmic artery (OA) Doppler has been described as an independent parameter in predicting PE [5, 7–9]. The OA is an easily accessible vessel, which provides information about the maternal arterial system. This insight is based on the concept of pulse wave (PW) propagation and reflection, a concept that is well established in hemodynamics. A PW is generated by left ventricular (LV) contraction and propagates to the cerebral vessels, generating a first systolic peak (P1), and along the arterial tree. During the transition to the periphery, reflection occurs; a reflection wave (RW) is built up, travels back along the aorta, and finally returns to the LV like an echo (Fig. 1) [10].

While passing the aortic arch, a fraction of the RW deviates cranially, re-accelerates cerebral blood flow, and thus generates a 2nd systolic velocity peak (P2) in cerebral Doppler waveforms [11–13]. Accordingly, P2 is the result of PW reflection and cranial transmission. As reflection intensity increases with vasoconstriction, the amplitude of P2 and the peak ratio PR = P2/P1 will increase accordingly [14].

Recent studies have shown that OA-Doppler assessment is a simple technique with good predictive performance for the development of PE [5, 7, 8, 15]. P2 and PR showed the best performance in prediction and complementary diagnosis of PE [7]. So far, OA-Doppler reference ranges have been established for the Brazilian population, but no reference ranges nor reference values exist for European pregnant women.

The aim of this study was to determine reference values for OA-Doppler indices in four representative 4-week study intervals: 1st, 2nd, and 3rd trimesters and at term in a mixed European population of low-risk pregnancies.

## Materials and methods

### Patients and study design

This is a prospective single-center cohort study, conducted between 2020 and 2024. Healthy pregnant women with singleton pregnancies, who accepted and signed informed consent, were included in the study. Women with a high risk for PE or who developed PE during pregnancy were excluded. A sample size calculation based on the precision of the estimated mean at four different time points during pregnancy led to the number of 240 pregnant women needed to establish reference values.

OA Doppler was performed according to a strict protocol and by trained staff members only. The Doppler was measured twice for each eye in a supine position. A linear transducer was placed transversely over the closed eye. Using the color flow imaging, the OA was identified by its direction of flow and pulsatility. Then, pulsed wave Doppler was applied placing the sample volume around 15–20 mm behind the optic disk, and medial to the optic nerve. The sample volume was 2–3 mm in length, since the maximal flow velocities in the OA range between 30 and 40 cm/sec and the data sampling and processing time of US Doppler systems ranges about 10 ms. In 10 ms, maximal flow moves around 3 mm, which is why the optimal SV size for OA Doppler is around 3 mm [16, 17]. The OA is insonated more or less longitudinally (insonation angle < 20°) and a minimum of three cycles was recorded. The ultrasound acoustic intensity settings were maintained according to the standards published by the safety group of the British Medical Ultrasound Society, with a thermal index of < 1.0 and a mechanical index of < 0.3 at all times [18].

For each woman, two measurements per eye were performed and the average value was included for the statistical analysis.

The measurements were performed at 4 distinct study intervals during pregnancy: 1st: 11–14 GW (gestational weeks), 2nd: 20–24 GW, 3rd: 30–34 GW, and 4th: at term, corresponding to the time points when low-risk pregnancies are routinely seen at our institution.

In total, 255 women were included in the study. Some women were assessed at more than one study interval. Therefore, the sum of all study observations exceeds the number of participants. For the 1st, 2nd, and 3rd trimesters and at term, 84, 74, 42, and 89 observations, respectively, were obtained.

As some participants contributed measurements at more than one study interval, analyses are considered exploratory and do not account for within-subject correlation across time points. Accordingly, the primary aim of the study was the descriptive generation of reference values rather than longitudinal modeling.

Also, recently published reference ranges from Brazil do not show substantial index changes in the gaps between these intervals [19].

**Fig. 2:**
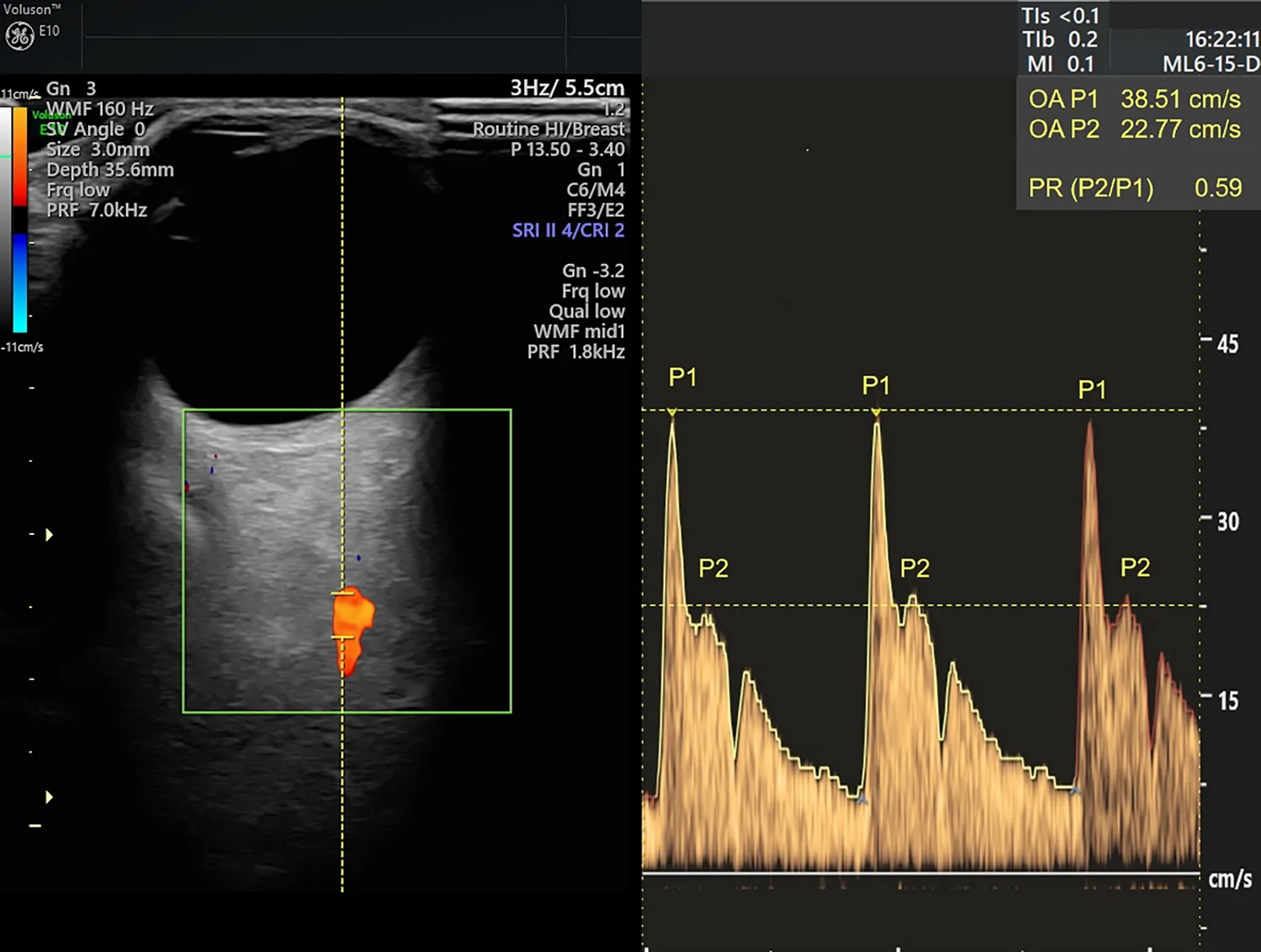
Sonography of the ophthalmic artery with corresponding flow velocity waveform. Left: identification of the OA medial to the optic nerve by color flow mapping (left). Right: typical OA-Doppler sonogram showing a biphasic systole with two systolic peaks, P1 and P2 (prior to the dicrotic notch)

Special focus was then led on the following systolic waveform parameters: P1, P2, and PR (Fig. 2). For these four selected measuring time points, reference values were generated for the different parameters by using the average of the two measurements of each eye. We only included measurements from low-risk pregnancies. Women with later on pregnancy complications were excluded.

### Data analysis

At the initial phase of the study, the Department of Biostatistics from the University of Zurich wrote the statistical analysis: In the first study phase, the research question was addressed and a sample size calculation for the derivation of normal values was done. For this analysis, existing evidence on the Doppler parameters as published in Brazilian studies was synthesized with meta-analysis techniques, resulting in a pooled mean estimate for peak ratio of 0.57 (95% CI). With a sample of 60 pregnant women at each of our chosen four time points, the resulting width of an estimated confidence interval for the mean peak ratio would be 0.057, which was chosen as 10% of the mean peak ratio.

Data coding and descriptive statistics were performed with SPSS version 26.0 (IBM, SPSS Inc., USA). Reference values are presented as mean ± standard deviation (SD) or median with interquartile range (IQR), depending on the distribution of the data. Further statistical analysis was performed using T test, Mann–Whitney *U* test, ANOVA, or Kruskal–Wallis test as appropriate. Additionally, a regression analysis was performed. Statistical significance was considered at *p* < 0.05.

Written informed consent was obtained from all women. The study was conducted under the principles of the Declaration of Helsinki and the International Conference on Harmonization E6 (Good Clinical Practice) guidelines as well as in accordance with the approval of the local Ethics Commission.

## Results

A total of 268 women were recruited for this study. Of these, 255 women were included in the analysis, as 11 cases (4%) were ruled out due to incomplete measurements of the OA Doppler and 2 (1%) were excluded as they developed PE later in pregnancy.

The baseline characteristics of this study population are presented in Table 1. Most women, 62%, were Caucasian, 7% Asian, 8% Afro-Caribbean, and 23% mixed or of unknown ethnicity. Mean maternal age was 33.9 ± 4 years. BMI was 22.5 (20.3–25) kg/m^2^.

**Table 1:** Maternal baseline characteristics (N = 255)

Baseline characteristics (*N* = 255)	
Maternal age (years)	33.9 ± 4
BMI (kg/m^2^)	22.5 (20.3–25)
Blood pressure (mmHg)	116 (108–124)/ 70 (65–76)
Ethnicity (%) • Caucasian • Asian • Afro-Caribbean • Mixed • Unknown	156 (62) 18 (7) 20 (8) 29 (11) 30 (12)

*Values are presented as mean ± SD (for maternal age), or median (with interquartile range, IQR, i.e., from 25 to 75th centile, for BMI and blood pressure), and N (%) (for ethnicity)

Median systolic and diastolic blood pressure (IQR) in the cohort was 116 (108–124)/70 (65–76) mmHg and remained stable from 11 GW with 117 (108–122)/68 (61–73) to 115 (108–120)/69 (65–76) mmHg near term.

The reference values for P1, P2, and PR averaged over the 4 study intervals were 33 cm/s (26–41 cm/s), 18.0 cm/s (14–22 cm/s), and 0.55 ± 0.14 for the right eye and 34.5 cm/s (27–42 cm/s), 19 cm/s (14–22 cm/s) and 0.56 ± 0.12 for the left eye (Table 2) with no significant difference between the right and left eyes.

**Table 2:** Comparison of the OA-Doppler values in the right and in the left eye

OA-Doppler values, averaged over the 4 GW intervals			
	Right	Left	*p *value
P1 (cm/s)	33 (26–41)	35 (27—42)	0.06
P2 (cm/s)	18 (14–22)	19 (14–22)	0.06
PR	0.55 ± 0.15	0.56 ± 0.12	0.12
avPR	0.559 ± 0.130		

*OA* Ophthalmic artery, *P1* first systolic peak, *P2* second systolic peak, *PR* peak ratio, *avPR* = PR left–right average value

The PR values at 11–14 GW, 20–24 GW, 30–34 GW, and at term are 0.56 ± 0.12, 0.54 ± 0.12, 0.52 ± 0.11, and 0.56 ± 0.11 for the right eye and 0.56 ± 0.12, 0.57 ± 0.11, 0.55 ± 0.12, and 0.57 ± 0.11 for the left eye, respectively, with no statistically significant differences between right and left eye (Table 3).

**Table 3:** OA-Doppler values during the distinct study intervals in the 1st, 2nd, 3rd trimesters, and at term

OA-Doppler values *right*					
	11–14 GW	20–24 GW	30–34 GW	Term	*p *value
P1 (cm/s)	32.1 (22.4–41)	35.1 (27.3–41.6)	32.2 (27.9–41.0)	33.8 (27.8–41.9)	0.21
P2 (cm/s)	18 (13–22.5)	17 (14.4–21.4)	18.5 (11.5–22.1)	18 (14–23)	0.42
PR	0.56 ± 0. 12	0.54 ± 0.12	0.52 ± 0.11	0.56 ± 0.11	0.06

OA-Doppler values *left*					
	11–14 GW	20–24 GW	30–34 GW	Term	*p *value
P1 (cm/s)	35.5 (26.6–40.8)	36.3 (28.6–43.4)	35.0 (27.5–45.0)	33.8 (27.5–40.5)	0.54
P2 (cm/s)	18 (12.5 -23.2)	18.8 (14.2–24.3)	19.8 (14.6—28)	19 (14.4–23)	0.66
PR	0.56 ± 0.12	0.57 ± 0.11	0.55 ± 0.12	0.57 ± 0.11	0.29

*OA* Ophthalmic artery, *P1* first systolic peak, *P2* second systolic peak, *PR* peak ratio

**Fig. 3:**
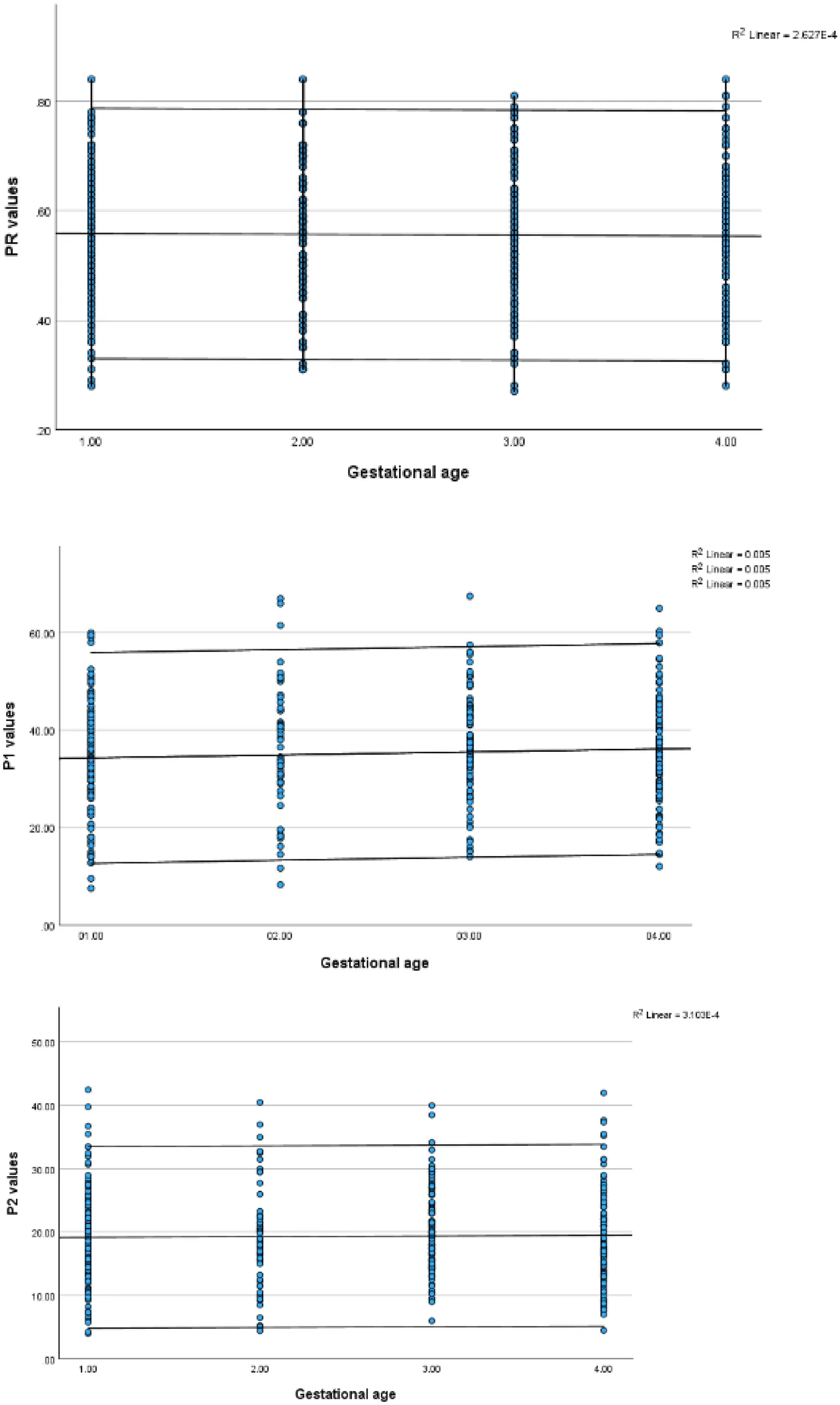
Scatter plots showing the Doppler values obtained in the 4 study intervals, 1–4, with regression lines of the measurements and the corresponding 95% CI. Doppler values: P1 and P2 values, in cm/s; PR values = P2/P1. Gestational age: study intervals no. 1: 11–14 GW; no. 2: 20–24 GW; no. 3: 30–34 GW, and no. 4: at term

Furthermore, P1 and P2 also remained stable among those four time points in pregnancy with no significant differences between the right and left eyes. An overview of the measurements of those four time points of pregnancy is listed in Table 3 and presented as scatter plots, as shown in Fig. 3. No significant differences were seen in Doppler values between the two eyes (*p* = 0.12) nor between the selected time points of measurement in pregnancy (*p* = 0.53).

No clinically relevant trend in PR values across gestational intervals was observed (Table 4).

**Table 4:** Regression analysis predicting PR among 4 time points (ANOVA overall model, regression coefficients)

Source	Sum of squares	Mean square	*F*	*p *value
Regression	0.093	0.023	1.726	0.144
Residual	3.771	0.014		
Total	3.865			

Variable	Beta	*t*	*p *value	95% CI lower	95% CI upper
(Constant)	–	6.569	< 0.001	0.378	0.702
1st trimester	0.099	0.310	0.757	− 0.138	0.190
2nd trimester	0.001	0.002	0.999	− 0.164	0.164
3rd trimester	− 0.085	− 0.334	0.739	− 0.194	0.138
term	0.072	0.218	0.828	− 0.146	0.182

## Discussion

This study presents OA-Doppler reference values for the systolic waveform parameters P1, P2, and PR at 4 study intervals: 11–14 GW, 20–24 GW, 30–34 GW, and at term, in healthy pregnant women of an ethnically diverse European tertiary-care population. These OA-Doppler parameters seem to be stable and comparable in a healthy pregnant population between those 4 time points, between the left and right eyes and between different ethnic groups. Under these physiological conditions, P2 can be expected to be approximately half as high as P1 on the OA-Doppler waveform (PR = 0.56 ± 0.13).

### OA-Doppler and reference values

Our results for P2 and PR for the right and left eyes are comparable to the results of other studies, mainly including reference values from Brazilian populations. De Oliveira et al. [21] established reference ranges for different OA-Doppler indices in singleton pregnancies between 20 and 40 GW. The only index without significant changes throughout normal pregnancy was the PR, with a mean PR of 0.53 (± 0.1) for the right eye *vs.* 0.56 (± 0.1) for the left eye [21]. In contrast to our study protocol, the measurements were performed once and not longitudinally throughout pregnancy. Alves et al. [22] published OA-Doppler reference values of normal pregnancies in the first trimester and found a slightly higher mean PR value: PR of 0.58 ± 0.11 [22]. In 2025, reference ranges were published for a low-risk pregnancy cohort from Brazil by Diniz et al. [19], as well as for Indonesia by Kusuma et al. [23]. Recently published reference ranges from Brazil do not show substantial index changes in the GA intervals between our 4 time points [19]. To our knowledge, no reference ranges nor values have been published for a European cohort up until today.

OA Doppler seems also valuable in hypertensive disorders of pregnancy (HDP), as PR values were shown to be higher in women with chronic hypertension (0.66 ± 0.14) or mild or severe PE (0.65 ± 0.10 or 0.89 ± 0.12) [21]. Sapantzoglou et al. [24] and the FMF (Fetal Medicine Foundation) London group found in a recently published paper that the inclusion of OA Doppler at the second screening improves the prediction of PE, especially preterm PE with delivery before 37 GW [24]. In several further studies, the FMF London group found clinical benefit in predicting preeclampsia by adding OA-Doppler measurements at several time points in pregnancy [15, 20, 24, 25]. In the meanwhile, OA Doppler was integrated in their PE risk assessment.

Practically, OA is an easily accessible vessel for color and Doppler flow assessment. The necessary ultrasound equipment is present in most obstetric units and delivery wards. We used a linear transducer, a transducer for breast sonography, which provides higher spatial and temporal resolution in the near field. However, standard transducers for fetal ultrasound are quite appropriate. The technique of OA-Doppler velocimetry may be easily learned by already trained prenatal sonographers, is well accepted by pregnant women, and is safe if performed within the recommended acoustic intensity limits [18].

### Global and local conditions of the maternal arterial system

Kalafat et al. [5] performed a systematic review and meta-analysis on OA Doppler for PE prediction. They found that OA Doppler has a standalone predictive value for developing early onset PE equivalent to that of uterine artery (UtA) Doppler evaluation [5]. In conclusion, they advocate efforts to elucidate the underlying mechanisms by which two seemingly unrelated maternal vessels can be used to predict PE. However, from a hemodynamic point of view, this is not surprising, because UtA and OA indices evaluate different biophysical conditions of the maternal arterial system: UtA PI evaluates downstream resistance in the uteroplacental circulation, i.e., a local condition, and OA PR evaluates vasoconstriction and PW reflection in the arterial system, i.e., a global condition. Both conditions show complementary changes even in the preclinical stage of PE, and thus, the combination of both may enhance PE prediction [14].

### PE and hemodynamic considerations

The observation that increased vasoconstriction in the arterial system leads to an increase in P2 in the OA is based on a study of Nakatsuka et al. [26]. They introduced the peak ratio PR = P2/P1 as a new Doppler index for the evaluation of the systolic OA-Doppler waveform, and made two key observations, namely a significant increase of PR with PE and a significant decrease of PR after administration of a vasodilator. Thus, it was evident that vasoconstriction of resistance vessels in the arterial system plays an essential role in PE and this effect can actually be visualized by OA Doppler.

In the meantime, it became obvious that vasoconstriction of peripheral resistance vessels and arterioles prevails in PE and, as such, causes stronger PW reflection than gestational or chronic hypertension. Thus, OA Doppler may distinguish between PE and other hypertensive disorders in pregnancy (HDP), even with the same degree of hypertension [25, 27].

### OA Doppler: diagnostic performance and cut-off

Diniz et al. [28] analyzed the Receiver-Operating Characteristic (ROC) curves of different OA-Doppler parameters and indices in a prospective study in order to identify the optimal parameter and the best cut-off value for complementary PE diagnosis. The best results were obtained with the PR and a cut-off value ≥ 0.75 (accuracy 87%, sensitivity 74%, and specificity 99%). This cut-off value corresponds very well to the value of 2 SD above the mean value of the PR reference ranges established by de Oliveira et al. [21]: PR + 2 SD ≈ 0.74 and also very well to mean PR + 1.645 SD (= 0.76) found in our study (agrees almost exactly with the 95th centile of our study population). However, cut-offs will need to be established for the European population in further studies.

Taking together all these above-discussed findings and considerations, OA-Doppler assessment seems a promising tool in predicting and diagnosing PE in the future.

### Limitations and strengths

To our knowledge, this is the first prospective cohort study, which generates OA-Doppler values in low-risk pregnant women in an ethnically diverse European cohort. It includes longitudinal and cross-sectional measurements. The primary aim of this study was descriptive generation of reference values rather than longitudinal modeling. Since some participants contributed measurements at more than one gestational interval, analyses are considered exploratory and do not account for within-subject correlation across time points. However, this approach to generate OA reference values may pave the way for further investigations on OA-Doppler assessment as a predictive and diagnostic tool regarding PE.

Further studies are needed to identify cut-off values for the prediction of PE and the administration of aspirin. Also, cut-off values for monitoring of women with PE and their use as a bedside tool to indicate appropriate medication or timely delivery are obviously still needed. Additionally, the value of OA Doppler for postpartum PE-monitoring and long-term surveillance beyond the postpartum period again seems promising but still needs further investigation.

## Conclusion

This study shows reference values of the OA-Doppler parameters (P1, P2, and PR) at 4 distinct GA intervals, for an ethnically diverse European tertiary-care population. These reference values are comparable with findings in other ethnicities, are stable during our four GA intervals, and do not significantly differ between left and right eyes. These results may pave the way for further investigations on OA-Doppler assessment as a predictive and diagnostic tool for PE.

## Supplementary Information

Below is the link to the electronic supplementary material.

Supplementary file1 (PNG 617 KB)

## Data Availability

The data that support the findings of this study are available on request from the corresponding author. The data are not publicly available due to privacy or ethical restrictions.
